# Supplementary material for: Knowledge and Treatment of Asymptomatic Hyperuricemia Versus Gout Among Physicians in Saudi Arabia: A Cross-Sectional Survey
Source: Healthcare (Basel). 2025 Oct 27;13(21):2719. doi: 10.3390/healthcare13212719 (PMC12607793; doi:10.3390/healthcare13212719)
Supplement: Supplementary file 1 [file healthcare-13-02719-s001.zip › healthcare-3893903-supplementary.pdf]

Table S1: Mapping of keyed practice and diet items

Item	Correct Key / Response	Guideline Source and Statement
Joint aspiration and analysis of synovial fluid for crystals	Correct	ACR 2020
Start anti-inflammatory drugs (colchicine, NSAID, or steroid) in combination with urate-lowering therapy at the same time	Correct (Conditional)	
Choice of anti-inflammatory (colchicine, NSAID, steroid)	Correct	
Continue anti-inflammatory prophylaxis for 3–6 months after starting ULT	Correct	
Indication: >1 gout flare per year	Correct	
Indication: First gout flare with CKD stage ≥3, tophi, or radiographic damage	Correct	
Indication: First flare with SUA >9 mg/dL or urolithiasis	Correct	
Allopurinol starting dose 100 mg once daily, titrate up	Correct	
Target serum urate <6 mg/dL (360 µmol/L)	Correct	
Continue ULT indefinitely with follow-up urate monitoring	Correct	
Organ meats high in purines (liver, kidney)	Avoid	EULAR 2016 and ACR 2012
High-fructose corn syrup–sweetened soda	Avoid	
Beef or lamb	Limit	ACR 2012 and Mayo Clinic
Seafood with high purine content (sardines, shellfish)	Limit	EULAR 2016 and Mayo Clinic
Alcohol overuse (>2/day men, >1/day women)	Avoid	EULAR 2016 and ACR 2020
Alcohol (small amount)	Limit	
Fruit juices high in fructose	Limit	EULAR 2016 and ACR 2012
Table sugar, sweetened beverages, desserts	Limit	ACR 2012 and Mayo Clinic
Table salt, including sauces	Limit	EULAR 2016 and Mayo Clinic

Low-fat or non-fat dairy	Encourage	EULAR 2016, ACR 2012, and Mayo Clinic
Vegetables	Encourage	EULAR 2016; Mayo Clinic
